# Epidemiology of Human and Animal Anthrax in India, 1990–2022: A Comprehensive Analysis of Literature and National Surveillance Data

**DOI:** 10.1155/bmri/5633425

**Published:** 2025-12-21

**Authors:** Suresh K. Puttahonnappa, Jessica Radzio-Basu, Hindol Maity, Ramya K. Rao, Robab Katani, Divakar Hemadri, Sharanagouda Patil, Jayashree Anand, Samer Singh, Divya Kandari, Rajinder Kaur, Rani Prameela, Shivraj Murag, Niranjana Sahoo, Vivek Kapur, Shah Hossain, Mohan Papanna

**Affiliations:** ^1^ National Institute of Veterionary Epidemiology and Disease Informatics, Bangalore, India; ^2^ Huck Institutes of the Life Sciences, Pennsylvania State University, University Park, Pennsylvania, USA, psu.edu; ^3^ Department of Microbiology, Mathma Gandhi Institute of Medical Sciences, Wardha, India; ^4^ Centre of Experimental Medicine & Surgery, Banaras Hindu University, Varanasi, India, bhu.ac.in; ^5^ Independent Researcher, New Delhi, India; ^6^ State Level Diagnostic Laboratory, Sri Venkateswara Veterinary University, Tirupati, India, svvu.edu.in; ^7^ Diagnostic Bacteriology and Mycology, SRDDL, Institute Of Veterinary Biological Animal & Health, Bangalore, India; ^8^ Department of Epidemiology and Preventive Medicine, Odisha University of Agriculture and Technology, Bhubaneswar, India; ^9^ Consultant, CisGEN Biotech Discoveries Pvt. Ltd, Chennai, India

**Keywords:** animal deaths, anthrax endemicity, *Bacillus anthracis*, cutaneous anthrax, intersectoral surveillance

## Abstract

**Background:**

Anthrax, a neglected zoonotic disease caused by *Bacillus anthracis*, exerts considerable health consequences in resource‐limited regions and is notably prevalent in India, causing persistent outbreaks that pose major animal and public health challenges. This study reviews the spatiotemporal patterns of human and animal anthrax outbreaks in India to identify high‐risk areas and assess the correlation with environmental factors.

**Methods:**

A comprehensive literature search covering the period from 1990 to 2022 was conducted across various databases including CAB Direct, PubMed, Scopus, and Web of Science, alongside Indian government databases like the Integrated Disease Surveillance Programme (IDSP) and the National Animal Disease Referral Expert System (NADRES). We extracted data from studies published in English, using predefined keywords, and evaluated them using the Joanna Briggs Institute checklists. Data analysis was carried out using Microsoft Excel and EpiInfo, with spatial mapping in ArcGIS Pro.

**Results:**

Out of the 423 studies reviewed, 44 fulfilled our inclusion criteria, providing data on 174 human outbreaks (1778 cases, 130 fatalities) and 1775 animal outbreaks (7818 deaths). We identified key hotspots for human anthrax in West Bengal, Odisha, and Andhra Pradesh, and significant hotspots for animal anthrax in Karnataka, Andhra Pradesh, Tamil Nadu, and West Bengal. Majority of human outbreaks were reported between March and June, whereas the majority of animal outbreaks were reported between June and September. A strong correlation was observed between rainfall and animal outbreaks in the eastern region (correlation coefficient of 0.94).

**Conclusion:**

The study highlights key hotspots for human and animal anthrax and discrepancies in human and animal anthrax reporting and gaps in surveillance. There is a critical need for enhanced One Health surveillance and animal anthrax vaccination programs for effective management and mitigate the disease. These strategies are essential not only for public health and livestock welfare in India but also for global health security.

## 1. Introduction

Anthrax, caused by the gram‐positive, endospore‐forming bacterium *Bacillus anthracis*, presents a neglected global health challenge [1]. While reported globally, the disease’s impact is disproportionately severe in low‐ and middle‐income countries, where limited resources, inadequate surveillance systems, and reduced access to vaccines exacerbate spread and severity.

Herbivores such as cattle, sheep, and goats are particularly susceptible. They contract anthrax through ingestion of *B. anthracis* spores in grazing areas, leading to systemic infections and often sudden death. In humans, anthrax is typically acquired through the consumption of contaminated meat or direct contact with infected animal products, which manifests in cutaneous, gastrointestinal, or inhalational forms. Untreated cutaneous anthrax, the most common form, can result in mortality in almost a quarter of cases [2]. The impact of anthrax is notably profound in South Asia, where a significant number of outbreaks are regularly reported [3, 4].

In India, for instance, large‐scale outbreaks in cattle and small ruminants pose a substantial risk to the large human populations nearby. A recent review focused on human cases from India indicates a high risk of human anthrax in specific regions such as Andhra Pradesh, Tamil Nadu, Karnataka, West Bengal, Odisha, and Jharkhand [5]. Nevertheless, significant gaps exist in understanding the occurrence of anthrax in animal and human populations, in addition to a poor understanding of regional distribution, and other contributing factors for anthrax in India.

Though anthrax is a reportable disease under both the National Animal Disease Referral Expert System (ICAR‐NADRES) [6] and the Integrated Disease Surveillance Program (IDSP) [7] in India, numerous challenges such as lack of awareness, geographical inaccessibility, and inadequate public health infrastructure, impede effective surveillance and reporting. This situation leads to an incomplete understanding of the disease epidemiology, high‐risk areas, and factors contributing to regular outbreaks. Therefore, we conducted a state‐of‐the‐art review of both academic and publicly available literature to address these gaps. This review was conducted with the specific aim of gaining a better understanding of anthrax epidemiology in India by analyzing spatiotemporal trends, identifying human and animal anthrax hotspots, examining the correlation between patterns of rainfall and anthrax outbreaks, and estimating the mortality rates due to anthrax in India between 1990 and 2022.

## 2. Material and Methods

This review follows the guidelines of Preferred Reporting Items for Systematic Reviews and Meta‐Analyses (PRISMA) [8]; however, due to the paucity of high‐quality data, a conventional systematic review and a meta‐analysis could not performed.

### 2.1. Search Strategy

We conducted a comprehensive search of multiple leading bibliographic databases, including CAB Direct, PubMed, BIOSIS, Scopus, and Web of Science between August and September, 2022 to capture relevant publications reporting anthrax between 2000 and 2022. We refined our search using MeSH terms for relevant publications using the following strings: *Human: (((Anthrax OR Anthracis) AND (human* ∗ *)) AND (India)) AND (epidemiolog* ∗). *Animal: ((((Anthrax OR “anthracis”) AND (India)) AND (Animal AND Livestock* ∗ *)) AND (Cattle OR Sheep OR Goat* ∗ *OR Buffalo OR Pig* ∗ *))) AND (Epidemiolog* ∗ *).* We also utilized reference chain searching to find peer‐reviewed publications. In addition, weekly outbreak reports between 2009 and 2022 were downloaded from the publicly accessible IDSP and NADRES, Government of India. Google and “Shodhganga: a reservoir of Indian theses” were searched on August 15, 2022, and September 7, 2022, respectively, to find additional sources that may have been missed. The screening of publications was guided by the inclusion and exclusion criteria specified in Table 1.

**Table 1:** Inclusion and exclusion criteria for selection of published studies.

**Inclusion**	**Exclusion**
Cross‐sectional studies, case series, correspondence, letter to editor, outbreak reports, and review articles	Newspaper articles or anecdotal evidence
Studies conducted in India	Studies conducted outside India
Studies reporting confirmation using microscopy, culture, or PCR including immunological assays for animal outbreaks	Studies or reports without laboratory confirmation either by microscopy, culture, immunological assays, or PCR
Publications between 1990‐2022	Any publication before 1990
Studies including anthrax data from humans and/or livestock and animals	Studies without data from human and/or livestock
English language	Non‐English
Full text of publication available	Full text unavailable
National reports on anthrax outbreaks validated by the government of India	Unvalidated reports

#### 2.1.1. Assessment of Methodological Quality

The articles were classified according to their study design and evaluated for the quality of their design, methodology, analysis, and interpretation. The Joanna Briggs Institute (JBI) critical appraisal checklists [9] were employed to assess the quality of these articles. We utilized JBI checklists designed for case reports, cross‐sectional studies cohort studies, and case–control studies. The quality of the animal studies was not evaluated due to the lack of standardized tools. Studies scoring above 70% on the JBI scale were classified as high quality. Those with scores between 50% and 70% were considered moderate quality, while scores below 50% were categorized as low quality.

### 2.2. Data Extraction and Analysis

Before data extraction, a template was developed based on demographics, clinical features, species, and other conditions common to anthrax. A feasibility test was conducted on 20 randomly selected articles to evaluate the inclusion and exclusion criteria. Based on this evaluation, data collection categories were finalized (Appendix Table 1).

Three team members (DK, SS, RR) independently reviewed titles, abstracts, and full‐text articles. JRB, RK, SH, and MP then subsequently reviewed these forms and removed duplicates. Discrepancies were resolved by collective discussions. Authors HM, SH, and MP reviewed all weekly IDSP data, extracted data on human anthrax outbreaks, and populated the data extraction form. The authors JRB and MP reviewed and finalized the data form. Due to the acute nature of anthrax presentations, our data extraction also included information from hospital‐based case series, case reports, and outbreak investigations. Authors KPS and MP compiled the animal outbreak data for India from 2001 to 2022 from the NADRES portal. Data from the first two years (2001–2002) during the establishment of the NADRES program were excluded as we suspected reporting bias (we observed an inexplicably high number of outbreaks reported). This report includes details from all recorded anthrax outbreaks in animal populations from 2003 to 2022.

#### 2.2.1. Primary Outcome

*Human Anthrax Case*: Defined as the number of laboratory‐confirmed (using microscopy, and/or culture, and/or PCR) cutaneous, inhalational, acute meningoencephalitis, septicemia, and gastrointestinal anthrax reported during the outbreak period. Data were sought for each type of anthrax during the outbreaks reported in peer‐reviewed publications and national reports.

*Animal Anthrax Cases*: Defined as the number of laboratory‐confirmed (using immunological assays, and/or microscopy, and/or culture, and/or PCR) animal deaths reported during the outbreak period. National animal or human outbreaks were considered to be confirmed by microscopy as the government laboratory network both under IDSP and NADRES confirm outbreaks by microscopy. If the outbreaks reported were between a time frame (e.g., 2014–2016), we considered them as at least one “outbreak” irrespective of human or animal mortality during the time frame, due to a lack of data on the number of outbreaks reported.

#### 2.2.2. Secondary Outcomes

*Case Fatality Rate* (CFR): The proportion of human anthrax cases that resulted in death and 95% confidence intervals (CIs) were calculated.

*Animal Death Rate:* The death rates for livestock species were calculated using the formula: [Number of animal deaths/Average animal population × 100,000] and 95% CIs were calculated.

Quantitative analysis was conducted using EpiInfo 7.2 software, and 2011 Indian census data were use to normalize maps (per million population). Heat maps were generated using ArcGIS Pro 3.1.2 software. The average district‐level animal populations were calculated using the 2019 and 2020 Livestock Census India. The plots representing the animal anthrax death rates with 95% CIs were created using Python. The monthly rainfall data from 1990 to 2022 was collected from The India Meteorological Department, Government of India [10]. The patterns of monthly rainfall data were superimposed on the overall national, south, and east regional human and animal anthrax outbreak curves, and the correlation between patterns of anthrax outbreaks and rainfall was tested using Spearman’s correlation coefficient “*r*” with 95% CIs and *p* values.

## 3. Results

**Figure 1:**
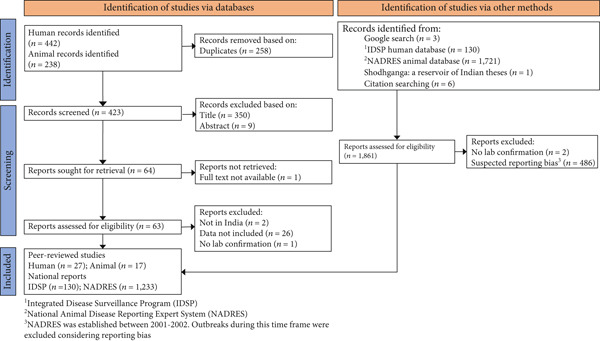
Schematic representation of the literature selection procedure. The publications were reviewed according to PRISMA guidelines. National reports from India were extracted from the IDSP weekly reports and NADRES monthly reports, and the full text of all publications meeting the criteria was evaluated.

A total of 680 articles were identified of which 442 human and 238 animal anthrax studies were identified. After removing duplicates, a total of 423 titles and abstracts were reviewed using the inclusion/exclusion criteria. From these, 63 reports were further assessed, 34 were included in the study, and an additional 10 studies were identified through a review of the citations in the selected reports. A total of 130 human anthrax reports were collected from the IDSP database, 1721 animal outbreaks from the NADRES database, and one from the Shodhganga website. In total, 1,363 national reports (IDSP and NADRES) and 44 studies from peer‐reviewed literature were included in the study (Figure 1, Table 2). From these sources, 174 human and 1,775 animal unique anthrax outbreaks were identified. The summary of the studies’ quality, characteristics, and risk of bias are presented in S2 Table.

**Table 2:** Summary of human and animal studies reporting anthrax.

**First author’s last name, year of publication**	**District (state)**	**Method of diagnosis**	**No. of human outbreaks**	**No. of animal outbreaks**
Human anthrax outbreaks				
George, S. et al. 1994 [11]	Chittoor (Andhra Pradesh)	LM/CUL	1	1
Lalitha, MK. et al. 1996 [12]	Arcot and Vellore (Tamil Nadu)	LM/CUL	1	
Kumar, A. et al. 2000 [13]	Puducherry	LM/CUL	3	
Thappa MD. et al. 2000 [14]	Puducherry	LM/CUL	2	
Datta, K.K. et al. 2002 [15]	Kolar (Karnataka) and Minapore (West Bengal)	LM/CUL/PCR	3	1
Sujatha, S. et al. 2002 [16]	Andhra Pradesh	LM/CUL	1	
Vijaykumar, M. et al. 2002 [17]	Puducherry and Villupuram, (Tamil Nadu)	LM/CUL	2	
Thappa MD. et al. 2003 [18]	Puducherry	LM/CUL	1	
Rao GRR. et al. 2005 [19]	Visakhapatnam (Andhra Pradesh)	LM/CUL/PCR	1	
Velayudhan, MN. et al., 2005 [20]	Villupuram (Tamil Nadu)	LM/CUL	1	
Rao GRR. et al. 2007 [21]	Visakhapatnam (Andhra Pradesh)	LM/CUL/PCR	1	
Bindu, M. et al., 2007 [22]	Tiruputi (Andhra Pradesh)	LM/CUL	1	
Narayan, S. et al. 2009 [23]	Villupuram and Tindivanam, (Tamil Nadu)	LM/CUL	2	
Rao TN, et al. 2009 [24]	Vizianagaram (Andhra Pradesh)	LM/CUL	1	
Ray TK, et al. 2009 [25]	Murshidabad (West Bengal)	LM	2	
David, S et al. 2010 [26]	Vellore (Tamil Nadu)	LM/CUL/PCR	1	1
Suggu, S et al. 2021 [27]	Visakhapatnam (Andhra Pradesh)	LM	1	1
Chakraborty PP et al. 2012 [28]	Midnapur West (West Bengal)	LM/CUL	1	
Reddy, R et al. 2012 [29]	Chittoor (Andhra Pradesh)	LM	1	1
Bhattacharya et al. 2013 [30]	Nadia (West Bengal)	LM/CUL	1	
Iqbal N et al. 2015 [31]	Puducherry	LM/CUL/PCR	1	
Mondal, TK et al. 2015 [32]	Purba Bardhaman (West Bengal)	LM/CUL	1	1
Deb, S et al., 2015 [33]	Burdwan (West Bengal)	LM/CUL	1	
Achar, A et al. 2018 [34]	Paschim Medinipur (West Bengal)	LM/CUL	1	1
Balachandrudu B et al. 2018 [35]	Visakhapatnam (Andhra Pradesh)	LM/CUL/PCR	1	
Garg N et al. 2018 [36]	Golaghat (Assam)	LM/CUL	1	
Kumar, M et al. 2019 [37]	Simdega (Jharkhand)	LM/CUL/PCR	1	
Nayak, P et al. 2019 [38]	Koraput (Odisha)	LM	8	1
Animal anthrax outbreaks				
Urs, MK et al. 1991 [39]	Davangere (Karnataka)	LM/CUL		1
Chaturvedi, VK et al. 2006 [40]	Jabalpur (Madhya Pradesh)	LM		1
Pandit, P K et al. 2006 [41]	Jalpaiguri (West Bengal)	LM		1
Venkatesha M et al. 2006 [42]	Chikballapur, Hassan, Kodagu, Kolar (Karnataka)	CFT		117
Murag, S et al. 2010 [43]	Chikballapur and Bangalore rurals (Karnataka)	LM/CUL/PCR		3
Patil RR et al. 2010 [44]	Bastar (Chhattisgarh)	LM		1
Suchitra, BR et al. 2010 [45]	Bengaluru Rural (Karnataka)	LM		1
Krithiga K et al. 2012 [46]	Thiruvananthapuram (Kerala)	LM		1
Yasothai, R et al. 2014 [47]	Erode (Tamil Nadu)	LM		1
Kingston JJ el al. 2015 [48]	Chamarajanagara (Karnataka)	LM/CUL/PCR		1
Malmarugan S et al. 2015 [49]	Tirunelveli (Tamil Nadu)	LM		2
Rajasokkappan, S. et al. 2016 [50]	Ramanathapuram (Tamil Nadu)	LM		14
Chandranaik et al. 2017 [51]	Bellary (Karnataka)	LM/CUL/PCR		1
Dandapat, P et al. 2017 [52]	West Midnapur (West Bengal)	LM/CUL/PCR	1	1
Chandranaik, B.M et al. 2020 [53]	Bellary (Karnataka)	LM/CUL/PCR		33
Roonie, A et al. 2020 [54]	Chamarajanagara (Karnataka)	LM/CUL/PCR		1
Singha, A et al. 2020 [55]	Nadia, Murshidabad, Hooghly (West Bengal)	LM/CUL/PCR		19
Total			44	207

Abbreviations: CFT, complement fixation test; CUL, culture; LM, light microscopy; PCR, polymerase chain reaction.

### 3.1. Temporal Trends of Human and Animal Anthrax Outbreaks in India 1990–2022

**Figure 2:**
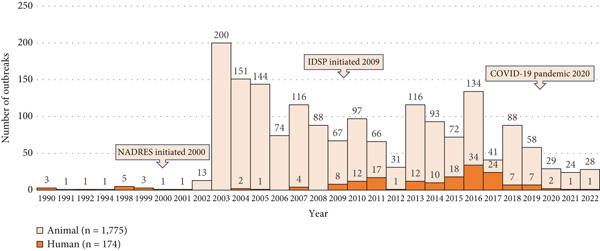
Temporal trends of human and animal outbreaks in India, 1990–2022. The number of animal anthrax outbreaks (light orange) and the number of human anthrax outbreaks (dark orange) are shown. Median animal outbreaks: 69, range 1–200, and median human outbreaks: 4, range 1–34.

The distribution of anthrax outbreaks by time indicates that either human or animal outbreaks were reported during most of the years spanning 1990–2022. The epidemiological curve shows sporadic outbreaks of animal and human outbreaks before 2000, following the implementation of NADRES. A median of 69 (range 1–200) animal outbreaks are reported annually. The human IDSP was initiated by the National Center for Disease Control in 2005, and converted into a national surveillance program in 2009. At this time, weekly outbreak reports were published, resulting in the reporting of a median of 4 (range 1–34) human anthrax outbreaks annually by IDSP. Notably, there was a sharp drop in animal and human anthrax reports from 2020, corresponding to the COVID‐19 pandemic (Figure 2).

### 3.2. Seasonal Trends of Human and Animal Anthrax Outbreaks in India

Animal anthrax outbreaks were observed year‐round (*n* = 1313), with the highest number of cases reported between June and October. In contrast, human outbreaks peak from March to September (*n* = 154; Figure 3a). A strong correlation exists between temporal patterns of animal anthrax outbreaks and rainfall (*r* = 0.82, 95% CI, 0.45 to 0.95, *p* < 0.001; Figure 3b). A weak correlation was observed between temporal patterns of human anthrax outbreaks and rainfall (*r* = 0.56, 95% CI, − 0.2 to 0.86, *p* < 0.05); however, most of the human outbreaks were observed between March and June (Figure 3b).

Figure 3Seasonal trends of human and animal anthrax outbreaks in India, 1990–2022. (a) The total number of animal (green, *n* = 1313), and human (orange, *n* = 154) anthrax outbreaks are shown by month. (b) The curves represent the patterns of animal and human outbreaks and their correlation with the rainfall pattern (blue). Correlation between patterns of animal outbreaks and rainfall Spearman’s correlation coefficient, *r* = 0.82 (95% CI, 0.45 to 0.95, *p* 0.001) and correlation between patterns of human outbreaks and rainfall Spearman’s correlation coefficient, *r* = 0.56 (95% CI, − 02 to 0.86, *p* 0.05).

**Figure:**
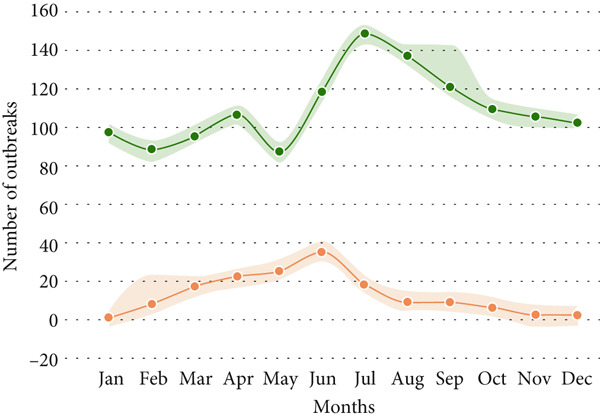
(a)

**Figure:**
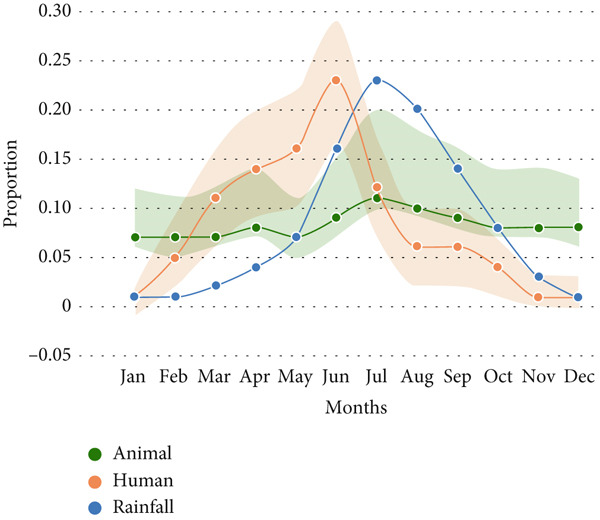
(b)

Furthermore, a strong correlation was observed between animal outbreaks and rainfall in the eastern region which includes the states of Assam, Odisha, Jharkhand, and West Bengal (Figure 4a; *r* = 0.94, 95% CI, 0.79 to 0.98, *p* < 0.0001). The outbreaks in this region start during the pre‐monsoon season (April) and reach a peak during the months of July and August, which coincides with the rainy season. Whereas there is no correlation between animal outbreaks and rainfall in the southern region, which includes the states of Karnataka, Andhra Pradesh, Kerala, Puducherry, and Tamil Nadu (Figure 4b; *r* = 0.35, 95% CI, − 0.29 to 0.77, *p* = 0.25).

Figure 4Seasonal trends of anthrax outbreaks in animal populations in east and south regions of India compared with rainfall in the regions, 1990–2022. Data from the included studies was parsed by region to determine if there are regional differences in the seasonal patterns of anthrax emergence in animal populations. (a) Correlation between patterns of animal outbreaks and rainfall in east, Spearman’s correlation coefficient, *r* = 0.94 (95% CI, 0.79 to 0.98, *p* 0.0001). (b) Correlation between patterns of human outbreaks and rainfall in South India, Spearman’s correlation coefficient, *r* = 0.35 (95% CI, − 0.29 to 0.77, *p* 0.25).

**Figure:**
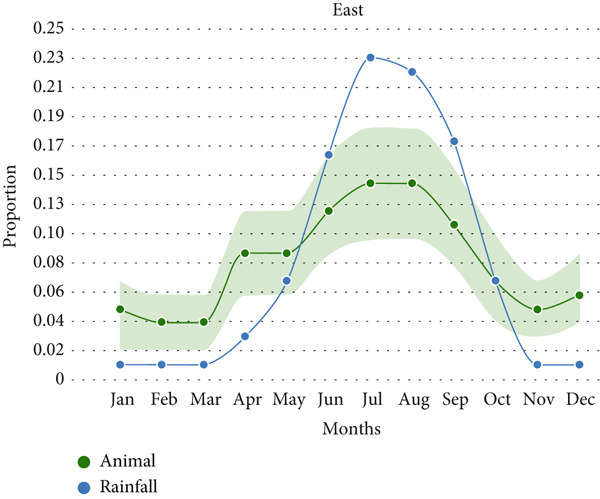
(a)

**Figure:**
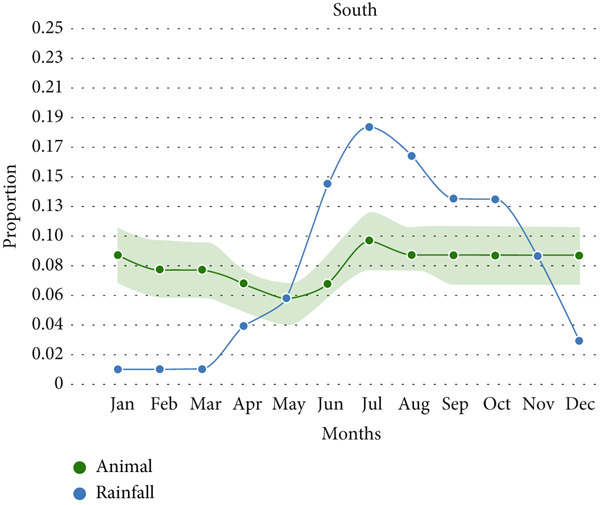
(b)

### 3.3. States and Districts Reporting Anthrax Outbreaks in India, 1990–2022

#### 3.3.1. Animal

A total of 16 states reported anthrax outbreaks in animals, and the states with the highest number of animal outbreaks included West Bengal (*n* = 186, 85 deaths/million ruminants), and Odisha (*n* = 97, 113 deaths/million ruminants) in the eastern region and Karnataka (*n* = 359, 303 deaths/million ruminants), Andhra Pradesh (*n* = 257, 219 deaths/million ruminants), and Kerala (*n* = 66, 115 deaths/million ruminants) in the southern region (Figure 5a,b, Tables 3 and 4).

Figure 5Geographic distribution of animal and human anthrax outbreaks by states in India, 1990–2022. Data from the included studies was parsed by region to determine if there are regional differences in the distribution of anthrax in animal and human populations. (a) The number of animal outbreaks by state. (b) The distribution of animal outbreaks per million cattle/sheep/goat population. (c) The number of human anthrax outbreaks by state. (d) The distribution of outbreaks per million population. Animal outbreaks are represented as shades of green color, and humans are represented as shades of orange color.

**Figure:**
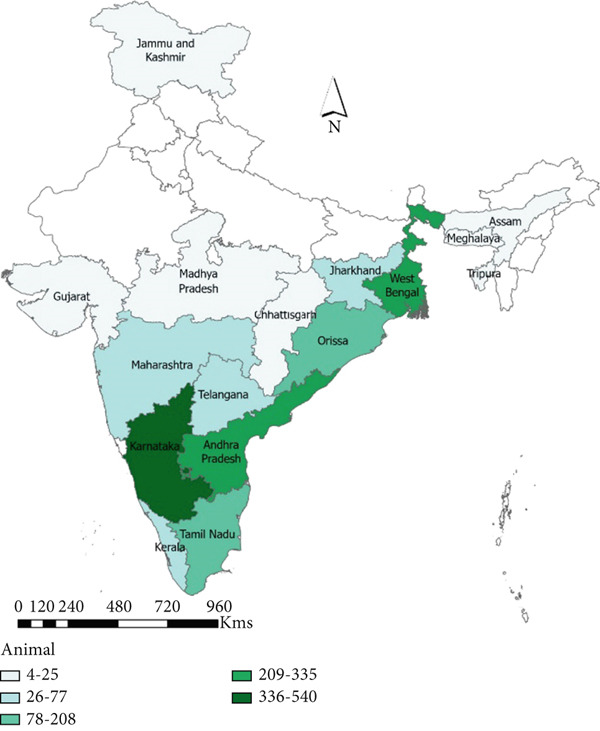
(a)

**Figure:**
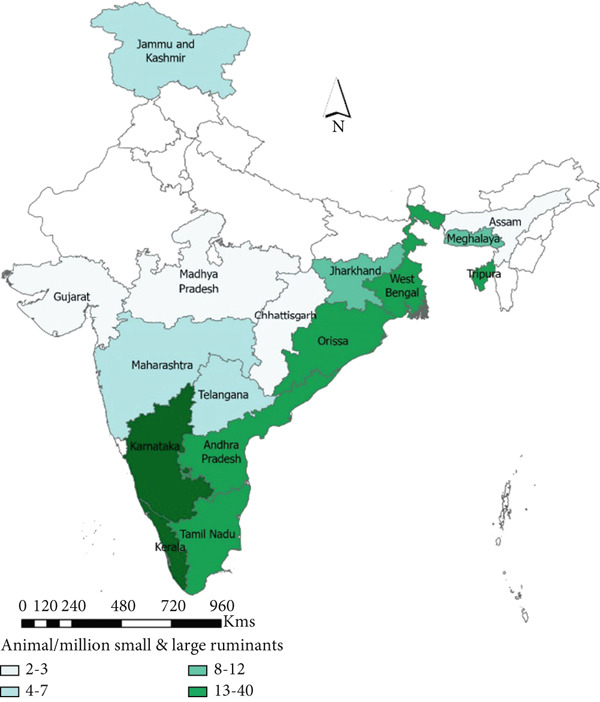
(b)

**Figure:**
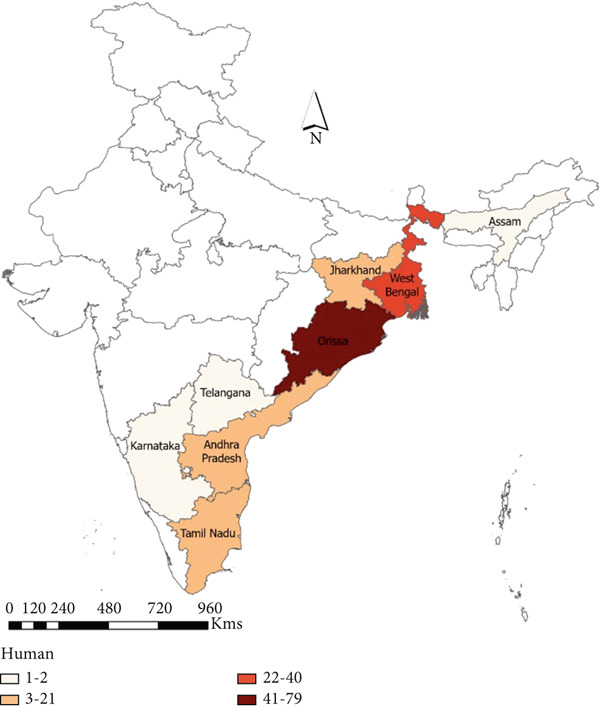
(c)

**Figure:**
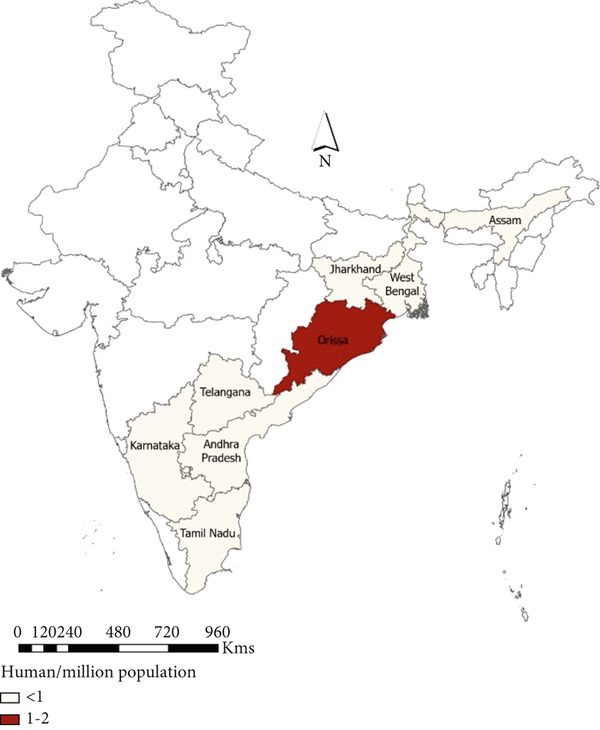
(d)

**Table 3:** State‐wise, human and animal anthrax outbreaks in India.

**Zone/state**	**Number of outbreaks (%)**		
	**Animal**	**Human**	**Total**
North			
Jammu and Kashmir	16 (1)	—	16 (1)
East			
Assam	12 (1)	1 (< 1)	13 (1)
Jharkhand	77 (4)	16 (9)	93 (5)
Odisha	128 (7)	79 (45)	207 (11)
Meghalaya	4 (< 0.5)	—	4 (< 0.5)
Tripura	6 (< 0.5)	—	6 (< 0.5)
West Bengal	247 (14)	40 (23)	287 (15)
Central India			
Chhattisgarh	6 (< 0.5)	—	6 (< 0.5)
Madhya Pradesh	25 (1)	—	25 (1)
West			
Gujarat	14 (1)	—	14 (1)
Maharashtra	46 (3)	—	46 (2)
South			
Andhra Pradesh	335 (19)	21 (13)	356 (18)
Karnataka	540 (27)	2 (1)	542 (28)
Kerala	69 (5)	—	69 (4)
Tamil Nadu and Puducherry	208 (12)	15 (9)	223 (11)
Telangana	57 (3)	1 (< 1)	58 (3)
Total	1775 (100)	174 (100)	1949 (100)

**Table 4:** State‐wise animal anthrax deaths and human anthrax case per million population.

**Zone/state**	**Animal deaths**	**Human cases**	**Average animal population (19 and 20 livestock census)**	**Human population**	**Animal anthrax deaths/million**	**Human anthrax cases/million**
North						
Jammu and Kashmir	26	—	2,620,445	—	10	—
East						
Assam	72	1	5,153,193	31,205,576	14	< 1
Jharkhand	70	142	6,333,408	32,988,134	11	4
Odisha	704	877	6,215,343	41,974,218	113	21
Meghalaya	30	—	388,459	—	77	—
Tripura	18	—	444,587	—	40	
West Bengal	893	465	10,481,923	91,276,115	85	5
Central India						
Chhattisgarh	26	—	3,647,090	—	7	—
Madhya Pradesh	81	—	9,453,402	—	9	—
West						
Gujarat	222	—	4,809,209	—	46	—
Maharashtra	297	—	8,903,771	—	33	—
South						
Andhra Pradesh	1544	133	8,384,924	84,580,777	219	2
Karnataka	2682	35	8,842,670	61,095,297	303	< 1
Kerala	101	—	879,798	—	115	—
Tamil Nadu and Puducherry	722	124	7,652,744	72,391,407	94	15
Telangana	330	1	8,455,444	35,003,674	4	< 1
Total	**7818**	**1778**	**92,666,410**	**415,511,534**	**84**	**4**

At the district level, outbreaks were reported across 176 districts between 1990 and 2022 (Figure S1A). The districts that frequently reported outbreaks included Murshidabad (43%, *n* = 80), Nadia 12% (*n* = 23), Barddhaman 11% (*n* = 20), and Koch Bihar 10% (*n* = 19) in West Bengal, and Koraput (*n* = 50) 51% and Sundergarh 13% (*n* = 13) in Odisha, eastern region. Bellary 18% (*n* = 67), Davanagere 14% (*n* = 51), Kolar 11% (*n* = 40), Koppal 11% (*n* = 39), and Tumkur 8% (*n* = 30) in Karnataka, and Chittoor 22.5% (*n* = 58), YSR Kadapa 21% (*n* = 55), Sri Potti Sriramalu Nellore 14% (*n* = 37), and Karnool 9% (*n* = 22) in Andhra Pradesh, southern region (Figure 6b,c, Appendix Table 2).

Figure 6District‐wise distribution of animal and human anthrax outbreaks in India, 1990–2022. (a) Anthrax endemic states in India. (b) Animal and human anthrax affected districts by states in the east. (c) Animal and human anthrax affected districts by states in the south. Animal outbreaks are represented in shades of green, and human outbreaks are represented in shades of orange. West Bengal (WB), Jharkhand (JH), Odisha (OD), Andhra Pradesh (AP), Telangana (TG), Karnataka (KA), Tamil Nadu (TN), and Kerala (KL).

**Figure:**
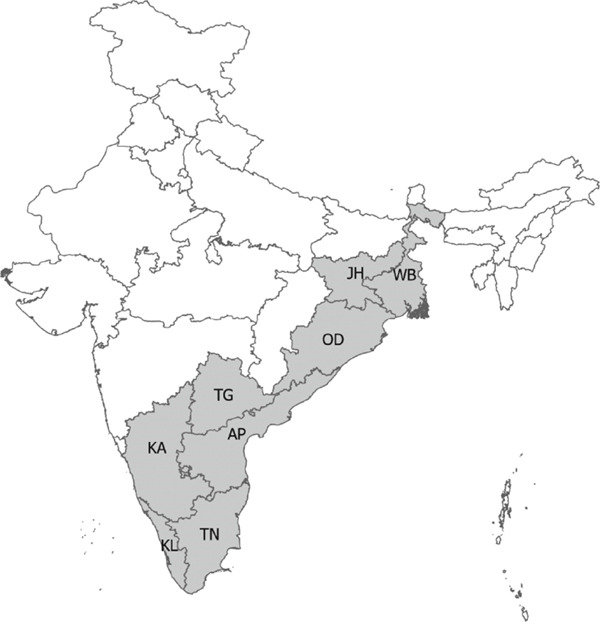
(a)

**Figure:**
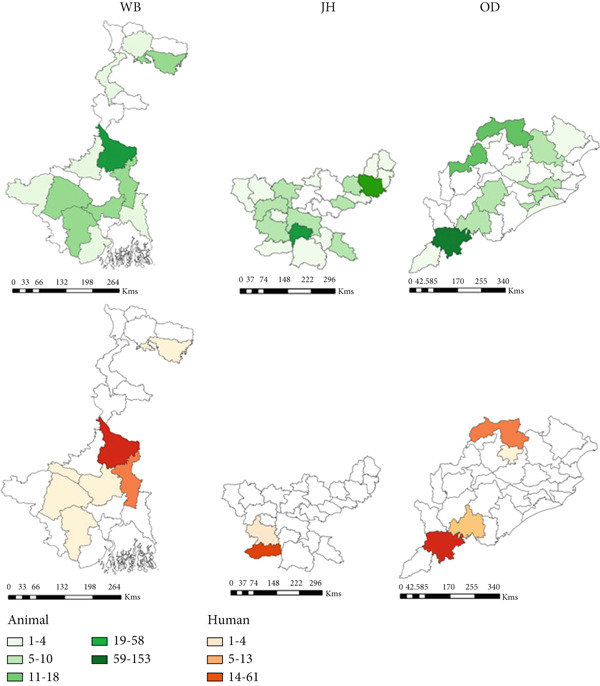
(b)

**Figure:**
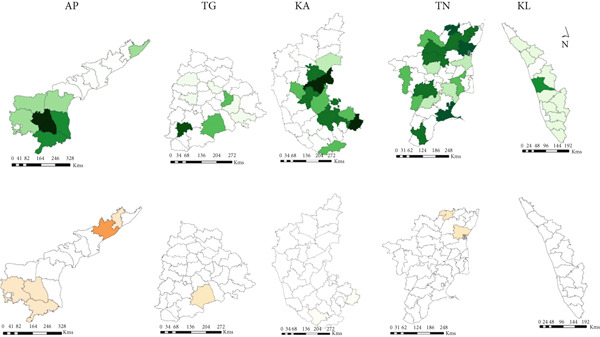
(c)

#### 3.3.2. Human

A total of eight states reported anthrax in humans (*n* = 174) (Figure 6a); these include the states of Odisha (45%, *n* = 77, 21 cases/million), West Bengal (23%, *n* = 40, 5 cases/million), Andhra Pradesh (13%, *n* = 21, 2 cases/million), and Tamil Nadu (14%, *n* = 8, 15 cases/million) with a few sporadic outbreaks from other parts of the country (Table 3 and Figure 5c,d). Human anthrax outbreaks occurred in a total of 29 districts between 1990 and 2022 (Figure S1B). The districts that frequently reported outbreaks include Murshidabad (*n* = 25), West Bengal, Simdega, Jharkhand (*n* = 16), Koraput (*n* = 59) and Sundergarh (*n* = 13), Odisha in the eastern region (Figure 6b), and Vishakhapatnam (*n* = 14), Andhra Pradesh in the southern region (Figure 6c).

### 3.4. Mortality Due to Anthrax in India

#### 3.4.1. Human

From 171 human outbreaks, a total of 1778 cases of human anthrax and 130 fatalities were recorded, resulting in an overall case fatality rate of 7% (95% CI, 6%–8%) in humans. Cutaneous anthrax was reported among 1630 (92%) individuals with 37 deaths, a CFR of 2% (95% CI, 1%–3%). Whereas other forms of anthrax or complications of anthrax (65 deaths out of 71 cases) included gastrointestinal (*n* = 12), acute meningoencephalitis (*n* = 51), and septicemia (*n* = 8), with an overall CFR of 91% (95% CI, 85%–98%).

#### 3.4.2. Animal

A total of 7657 deaths were reported in animals between 1990 and 2022. The major livestock species affected include sheep (47%), bovine (39%), goats (5%), pigs (1%), buffalo (< 1%), sheep and bovine/goat/buffalo (6%), and wild animals (1%) such as deer, elephants, rhinoceroses, hyenas, rabbits, and wild boar (Table S1).

### 3.5. District‐Wise Animal Anthrax Death Rate per 100,000 Animal Population by States in India, 2003–2022

A total of 1,328 animal outbreaks occurred between 2003 and 2022. We have presented the death rates in high‐burden districts (defined as at least 10 deaths/100,000 animals). Full details on the mortality rates are available in Appendix Table 2.

#### 3.5.1. East

##### 3.5.1.1. West Bengal

Murshidabad 24.2 (95% CI, 21.2–27.1) and Koch Bihar 12.7 (95% CI, 10.6–14.9) districts reported high death rates, mainly in cattle (Figure S2A).

##### 3.5.1.2. Odisha

Koraput 52.6 (95% CI, 45.9–59.2), Bargarh 21.2 (15.9–26.5), Sundergarh 16.2 (12.8–19.7), and Rayagada 10.8 (6.9–14.8) districts reported high death rates in cattle. Meanwhile, Koraput also reported high death rates among sheep, 38.0 (27.2–48.9), and goats, 45.6 (34.9–56.4); Figure S2A.

#### 3.5.2. South

##### 3.5.2.1. Andhra Pradesh

Anthrax death rates in cattle were 15.3 (95% CI, 11.6–19.0) and 11.8 (95% CI, 9.6–14.0) in the Vizianagram and Chittoor districts, respectively. YSR Kadapa had the highest death rates (22.9, 95% CI, 20.6–25.2) in sheep, followed by Karnool 10.8 (95% CI, 9.2–12.3) and Nellore 10.2 (95% CI, 8.4–12.0). Srikakulam reported the highest death rates in goats (20.1 [95% CI, 14.8–25.4] (Figure S2B).

##### 3.5.2.2. Karnataka

High anthrax death rates in cattle were observed in Davanagere (38.0, 95% CI, 32–44.0), Kolar (28.5, 21.8–35.2), Kodagu (27.3, 16.4–38.3), Bangalore rural (22.0, 15.1–29.0), and Chamarajanagar (19.9, 95% CI, 14.5–25.3). The districts of Raichur (75.1, 95% CI, 68.4–84.7), Bellary (67.7, 95% CI, 62.2–73.1), Davanagere (35.1, 95% CI, 29.5–40.7), and Koppal (29.2, 24.8–33.5) had high death rates in the sheep (Figure S2B).

##### 3.5.2.3. Tamil Nadu

Dharmapuri (20.1, 95% CI, 15.3–24.9), Theni (12.0, 95% CI, 5.5–18.5), and Vellore (11.4, 95% CI, 8.6–14.2) districts reported high death rates in cattle. Whereas Thanjavur (83.0, 95% CI, 51.7–114.3), Madurai (19.1, 95% CI, 12.0–26.2), Kanchipuram (18.5, 95% CI, 10.7–26.2), Coimbatore (12.4, 95% CI, 5.7–19.2), and Dindigul 12.2 (95% CI, 5.8–18.7) districts reported high death rates in sheep (Figure S2B).

### 3.6. Laboratory Diagnosis of Anthrax Outbreaks

Of the outbreaks reported in the literature, we observed that 21% (9/44) of human and 60% (57/95) of animal outbreaks were confirmed by culture and/or PCR, respectively. Microscopy was the main method (43%) of confirmation in humans, and the majority of the outbreak reports by IDSP and NADRES were confirmed using microscopy (Table 5).

**Table 5:** Methods used for laboratory confirmation of anthrax in peer‐reviewed studies.

**Laboratory diagnostics**	**Human outbreaks (*n* [%])**	**Animal outbreaks (*n* [%])**
Microscopy	19 (43)	16 (17)
Microscopy/culture	16 (36)	2 (2)
Culture/PCR	9 (21)	57 (60)
Complement fixation	0 (0)	20 (21)
Total	44 (100)	95 (100)

## 4. Discussion

To elucidate the spatiotemporal dynamics of anthrax in humans and animals in India, we collected data from well‐known databases, online search engines, and national reporting systems. After reviewing 44 selected peer‐reviewed articles and 1363 national outbreak reports from these sources, we extracted data on a total of 171 human anthrax outbreaks and 1328 animal anthrax outbreaks occurring between 1990 and 2022. Analyzing this dataset enabled us to identify key patterns and trends in anthrax occurrence across India. Our findings reveal that animal anthrax outbreaks are reported year‐round, whereas human outbreaks were primarily observed from March to August. The south and eastern region states such as Andhra Pradesh, Jharkhand, Odisha, Tamil Nadu, and West Bengal reported the highest number of human anthrax cases. At the same time, Karnataka, Andhra Pradesh, Tamil Nadu, Odisha, and West Bengal were most affected by animal anthrax outbreaks.

A significant correlation was observed between animal anthrax outbreaks and the rainy season (June to September) in the eastern region, contrasting with the southern region, where outbreaks were reported throughout the year, with a slight peak during the rainy season. The human anthrax outbreaks did not show a correlation with rainfall. Instead, most human outbreaks were observed during the pre‐monsoon or early part of the rainy season (March to June), followed by a sharp decline in the number of outbreaks. This trend aligns with seasonal patterns reported in a previous study from Eastern India [38]. Although it is widely recognized that human anthrax cases stem from exposure to infected animals or their products, these discrepant findings between the occurrence of human outbreaks followed by animal outbreaks emphasize the gaps in disease reporting by animal health sectors in India. This highlights the need for enhanced intersectoral collaboration and the need for strengthening the animal health surveillance system. Further, we noted that states with high levels of animal anthrax reported few or no corresponding human outbreaks. For example, Karnataka, with a high incidence of animal anthrax (27% of total reported cases), reported only two sporadic cases of human anthrax in about two decades. In contrast, states like Odisha and West Bengal, which frequently reported human anthrax outbreaks (45% and 23% of total reported cases, respectively), accounted for only 7% and 14% of reported animal outbreaks, respectively. These findings suggest that the observed differences in animal and human anthrax incidence across states may be multifactoral, such as weaknesses in disease surveillance and reporting systems, shortage of trained manpower, limited laboratory capacity and infrastructure, and disparities in healthcare access and treatment‐seeking behavior among affected populations. Further research is needed to identify the most significant contributors to these patterns and to guide targeted interventions for anthrax control and prevention in both animals and humans.

An analysis of the district‐level data identified several anthrax hotspots. In southern India, districts sharing borders between Karnataka (Raichur, Bellary, Koppal, Davanagere, Chitradurga, Tumkur, and Kolar) and Andhra Pradesh (Karnool, Chittoor, Anantapur, YSR Kadapa, Prakasam, and Sri Potti Sriramalu Nellore) were identified as key hotspots for animal anthrax. However, corresponding human outbreaks in these areas were relatively scarce. In the eastern region, Murshidabad and neighboring districts in West Bengal bordering anthrax‐endemic districts of Bangladesh (Pabna, Sirajganj, Rajshahi, Kushtia, and Tangail), indicating the possibility of transboundary spillover risk of anthrax [4]. Additionally, Simdega district in Jharkhand, which borders the anthrax‐endemic district of Sundergarh in northern Odisha. Koraput district in southern Odisha, which reported the highest number of human anthrax cases in India, is the hotspot for human anthrax. Apart from the aforementioned anthrax‐endemic states, animal anthrax outbreaks were also reported in Assam, Jammu Kashmir, Gujarat, Maharashtra, Madhya Pradesh, Chhattisgarh, Telangana, and Kerala. Although no documented human anthrax cases have been reported from these states, a recent human anthrax predictive risk map [5] aligns with these states, indicating a potential risk of disease spillover to humans. These findings suggest the presence of well‐defined anthrax hotspots, emphasizing the need for detailed studies to elucidate factors contributing to anthrax endemicity in these areas, such as animal migration, soil and environmental conditions, carcass disposal practices, and the utilization of animal anthrax vaccination.

The US Centers for Disease Control and Prevention (CDC) states that untreated anthrax has mortality rates of 20% for cutaneous, 60% for gastrointestinal, and 100% for pulmonary forms [2]. A recent systematic review looking at human mortality factors in India found mortality rates ranging from 2% to 38% for cutaneous anthrax and 100% for other forms [5]. Our study identified a total of 1778 cases of human anthrax, leading to 130 fatalities across 29 districts. The predominant form of the disease was cutaneous anthrax with a mortality rate of 2%. In contrast, gastrointestinal anthrax and other anthrax complications had a significantly higher mortality rate of 92%. These findings highlight the risk to public health posed by anthrax in endemic districts.

In the livestock sector, there were 7657 fatalities across 176 districts, resulting in death rates ranging between 1 and 83 per 100,000 animals. Sheep (47%) and cattle (39%) were the most affected species, followed by goats (5%). The reasons for the differential susceptibility and resulting death rates among these species are not yet fully understood. However, it could be hypothesized that their distinct grazing habits affect their exposure to anthrax spores. Sheep and cattle, which graze closer to the ground, may face higher risks of encountering these spores compared to goats, which graze less and browse more at elevated levels, potentially reducing their exposure [56]. Furthermore, cattle are often used for plowing fields in endemic areas, increasing their risk of exposure to anthrax spores. Sheep may be at increased risk of missing anthrax vaccination due to their short production turnover driven by high market demand for meat, as well as livestock owners’ decisions not to vaccinate. In endemic districts of Odisha, such as Koraput, where cattle populations are substantially higher compared to sheep and goat populations resulting in morefreuent anthrax outbreaks among cattle [10]. Other key determinants may be associated with limited community acceptance of vaccination, irregularities in vaccine supply, and insufficient availability of trained vaccinators. The observed differences in anthrax incidence and mortality across livestock species highlight several critical programmatic gaps that warrant urgent attention. Strengthening species‐specific surveillance systems is essential to ensure more accurate capture of anthrax cases in both large and small ruminants. Enhanced outbreak investigations at the district level would also provide valuable insights into species‐specific vulnerabilities and guide targeted interventions. Investments in building diagnostic capacity, trained manpower, and risk‐based vaccination strategies are critical to reducing recurrent outbreaks in endemic regions.

The World Organization for Animal Health (WOAH) and the World Health Organization (WHO) recommend PCR methodologies targeting genes in the pXO1 and pXO2 plasmids as the standard for anthrax diagnosis [1]. However, our study found that microscopy remained the predominant diagnostic method within the government health and animal husbandry sectors in India, which suggests a lack of access to suitable diagnostics like PCR in endemic areas. To address such issues, the Government of India has recently implemented several initiatives to enhance laboratory surveillance. One significant initiative is the Integrated Health Information Platform (IHIP), developed in collaboration with WHO following the joint monitoring mission IDSP report in 2015 [57]. IHIP‐IDSP has prioritized 33 diseases, including anthrax, for surveillance in India. This initiative, along with other efforts to strengthen surveillance in the animal health sector (https://dahd.nic.in/animal-pandemic-preparedness-initiative), aims to integrate national surveillance systems and enhance laboratory‐based surveillance. Post‐COVID‐19, India has developed PCR diagnostic capabilities at District Public Health Laboratories (DPHLs) under IDSP, which could be utilized for anthrax diagnosis in endemic areas, aiding in rapid outbreak response.

## 5. Limitations

This study provides a comprehensive overview of the anthrax situation in both human and animal health sectors, highlighting major gaps. However, the study’s limitations include the scanty data from systematically conducted cross‐sectional or observational studies. As most of the available data in peer‐reviewed publications or anthrax surveillance were either case reports, case series, and a handful of observational studies, or outbreak reports, describing individual or small groups of patients without using control groups or comparison arms. This lack of controlled data makes it difficult to establish causality or generalize findings beyond the specific cases presented.

## 6. Conclusion

This study elucidates the complex dynamics of anthrax in India, emphasizing significant gaps in surveillance and reporting. It highlights the importance of enhanced intersectoral collaboration, standardized diagnostic practices, and targeted research to understand the factors contributing to anthrax endemicity. The findings suggest an urgent need to integrate human and animal health surveillance systems, utilize existing laboratory capacities for improved diagnosis, and conduct detailed studies on environmental and animal husbandry practices influencing anthrax transmission. This approach is essential for developing effective prevention and control strategies against anthrax, safeguarding public health and livestock welfare in India, and contributing to broader global health security.

### 6.1. Strategic Approach to Anthrax Prevention and Control in Endemic Regions in India

Our review and observations indicate that anthrax prevention and control in India face multiple challenges, including limited understanding of disease dynamics, inadequate infrastructure, sociocultural factors, and insufficient intersectoral coordination. To address these issues, we propose a strategic framework for effective anthrax control. Key inputs include enhancing infrastructure, ensuring a trained workforce, strengthening intersectoral communication and outbreak response, providing standardized diagnostics, maintaining a steady supply of diagnostics and animal anthrax vaccines, conducting risk mapping, studying local disease patterns, and improving risk communication. These efforts will result in establishing a robust intersectoral surveillance system to generate timely data, implement routine animal anthrax vaccination, and promote safe carcass disposal practices to prevent anthrax spillover from animals or the environment to humans (Figure S3).

## Conflicts of Interest

The authors declare no conflicts of interest.

## Author Contributions

**Conceptualization:** Mohan Papanna, Jessica Radzio‐Basu, Shah Hossain, and Suresh K Puttahonnappa. **Data curation:** Hindol Maity, Ramya K Rao, Samer Singh, Divya Kandari, Rajinder Kaur, Jayashree Anand, and Robab Katani. **Constructed the methodology:** Mohan Papanna, Suresh K. Puttahonnappa, Shah Hossain, and Jessica Radzio‐Basu. **Formal analysis:** Mohan Papanna and Jessica Radzio‐Basu. **Writing—original draft:** Mohan Papanna, Jessica Radzio‐Basu, Shah Hossain, Robab Katani, and Suresh K. Puttahonnappa. **Writing—review and editing:** Divakar Hemadri, Sharanagouda Patil, Rani Prameela, Shivraj Murag, Niranjana Sahoo, and Vivek Kapur. Suresh K. Puttahonnappa and Jessica Radzio‐Basu contributed equally to this work.

## Funding

This study is supported by the DTRA‐Biological Threat Reduction Program, HDTRA1‐21‐1‐0036.

## Supporting Information

Additional supporting information can be found online in the Supporting Information section.

## Supporting information


**Supporting Information 1** Table S1: Species‐wise mortality due to anthrax. Table S2: Assessment of study quality, characteristics, and risk of bias.

**Supporting Information 2** Figure S1: Overall district‐wise distribution of animal and human anthrax outbreaks in India, 1990–2022. Figure S2: Death rates due to anthrax in small and large ruminants per 100,000, 2003–2022. (a) Animal anthrax death rates in east. (b) Animal anthrax death rates in south. Death rates for cattle (blue), sheep (red), and goat (black), and the 95% confidence intervals are represented. Figure S3: Strategic approach to establish capability and improve anthrax prevention and control in endemic regions in India.

**Supporting Information 3** Appendix 1: Overall study data included in the analysis, stratified by relevant variables in columns, and rows contain the date related to individual human and animal outbreaks.

**Supporting Information 4** Appendix 2: Data used to calculate district‐level animal anthrax death rates and 95% confidence intervals for cattle, sheep, and goat.
