# Supplementary material for: Epidemiology of Human and Animal Anthrax in India, 1990–2022: A Comprehensive Analysis of Literature and National Surveillance Data
Source: Biomed Res Int. 2025 Dec 21;2025:5633425. doi: 10.1155/bmri/5633425 (PMC12719797; doi:10.1155/bmri/5633425)
Supplement: Supplementary file 1 — Supporting Information 1 Table S1: Species‐wise mortality due to anthrax. Table S2: Assessment of study quality, characteristics, and risk of bias. [file BMRI-2025-5633425-s003.docx]

**Supplemental 1 Table: Species-wise mortality due to anthrax**

| **Species** | **Deaths n(%)** |
| --- | --- |
| **Human** | 130 (2) |
| **Sheep** | 3,681 (46) |
| **Bovine** | 3,129 (39) |
| **Goat** | 291 (5) |
| **Pig** | 100 (1) |
| **Buffalo** | 63 (1) |
| **Mixed species*** | 217 (6) |
| **Wildlife** | 32 (1) |
| **Total** | 7,787 |

^*^Sheep/bovine or Sheep /goat or Sheep/buffalo

**Supplementary 2 Table: Assessment of study quality, characteristics and risk of bias**

| **First author's last name, year of publication** | **Study design** | **Tool used** | **Study quality rating** | **Characteristics and Risk of Bias** |
| --- | --- | --- | --- | --- |
| George, S. et al. 1994^[13]^ | Case series | JBI critical appraisal checklist | 7/8 (88%) | Study describes an anthrax meningoencephalitis outbreak in Chittoor, India, linked to handling a sheep carcass, resulting in several deaths. The study may be biased due to its focus on a localized incident and lacks advanced diagnostic confirmation |
| Lalitha, MK. et al. 1996^[14]^ | Case series (Correspondence) | JBI critical appraisal checklist | 4/8 (50%) | Study focuses on endemic anthrax in southern India, noting an increase in human cases linked to better disease awareness and reporting. It faces potential biases from focusing on known endemic areas and challenges due to under-reporting and limited diagnostic facilities. |
| Kumar, A. et al. 2000^[15]^ | Case series (Correspondence) | JBI critical appraisal checklist | 4/8 (50%) | The study uses a well-defined study population and employs standardized measures but may be biased due to unadjusted confounders and has limited generalizability due to its narrow focus on a specific group and lacks advanced diagnostic confirmation |
| Thappa MD. et al. 2000^[16]^ | Case series | JBI critical appraisal checklist | 6/8 (75%) | The study reports cutaneous anthrax, emphasizing the clinical presentation and treatment outcomes. It is a hospital-based study affected by selection bias. The study reflects regional health practices, potentially limiting the generalizability of the findings. |
| Datta, K.K. et al 2002^[17]^ | Case series | JBI critical appraisal checklist | 4/8 (50%) | This study has potential bias due to the focus on reported cases and outbreaks with limited epidemiological data |
| Sujatha, S. et al. 2002^[18]^ | Case report | JBI critical appraisal checklist | 8/8 (100%) | The study reports a rare case of anthrax peritonitis in a pregnant women emphasizing the clinical presentation and treatment outcomes. |
| Vijaykumar, M. et al. 2002^[19]^ | Case series | JBI critical appraisal checklist | 5/8 (63%) | The study discusses cutaneous anthrax in South India, noting a higher incidence in children. It may have biases due to its data being sourced from a single institution, potentially limiting wider applicability. |
| Thappa MD. et al. 2003^[20]^ | Case report | JBI critical appraisal checklist | 7/8 (88%) | The case report details a young female in India diagnosed with cutaneous anthrax of the eyelid, confirmed via Gram stain but not culture. The study may be biased due to reliance on clinical diagnosis, lacks advanced diagnostic confirmation and epidemiological data. |
| Rao GRR. et al. 2005^[21]^ | Case series | JBI critical appraisal checklist | 7/8 (88%) | The study details a cutaneous anthrax outbreak in Vizianagaram, India, diagnosed through clinical and basic microbiological methods. It may be biased due to its focus on a specific tribal region and lacks advanced diagnostic confirmation |
| Velayudhan, MN. et al., 2005^[22]^ | Case report | JBI critical appraisal checklist | 7/8 (88%) | The study describes a rare case of inhalational anthrax causing ARDS in a child, highlighting rapid progression and treatment challenges. The findings may be limited in broader applicability due to the focus on a single case. |
| Rao GRR. et al. 2007^[23]^ | Case report | JBI critical appraisal checklist | 7/8 (88%) | The study focuses on cutaneous anthrax in a tribal area of Visakhapatnam, revealing that direct contact with infected animals was a common transmission route. The potential bias includes the study's reliance on self-reported data for identifying risk factors and lacks advanced diagnostic confirmation |
| Bindu, M. et al., 2007^[24]^ | Case report | JBI critical appraisal checklist | 7/8 (88%) | The study reports a case of anthrax meningoencephalitis emphasizing the clinical presentation and treatment outcomes |
| Narayan, S. et al. 2009^[25]^ | Cross sectional study | JBI critical appraisal checklist | 6/9 (67%) | The study is limited by its retrospective nature and the small number of cases, which may not fully capture the disease's dynamics or the effectiveness of interventions over a wider area or varied populations. |
| Rao TN , et al. 2009^[26]^ | Case report | JBI critical appraisal checklist | 5/8 (63%) | Study cutaneous anthrax among four tribal men in Andhra Pradesh, confirmed by traditional lab tests after contact with infected animals. Its conclusions may be limited due to the small sample size and the lack of advanced molecular testing. |
| Ray TK, et al. 2009^[27]^ | Cohort study | JBI critical appraisal checklist | 7/11 (64%) | The study investigates cutaneous anthrax outbreaks in West Bengal, showing high attack rates associated with handling and consumption of meat from slaughtered cattle. Bias might arise from the retrospective cohort study design, relying on self-reported data, which could affect the accuracy and completeness of exposure and outcome information. |
| David, S et al. 2010^[28]^ | Case report | JBI critical appraisal checklist | 8/8 (100%) | The study describes a case of oculocutaneous anthrax confirmed by PCR in a patient exposed to a dead sheep, emphasizing the importance of prompt antibiotic treatment. The findings may have limited generalizability due to reliance on a single case. |
| Suggu, S et al. 2021^[23]^ | Case report | JBI critical appraisal checklist | 7/8 (88%) | The study reports a case of cutaneous anthrax in a tribal man from Araku Valley, confirmed by PCR. Potential biases stem from its focus on a small, specific group, limiting broader applicability |
| Chakraborty PP et al. 2012^[29]^ | Cross sectional study | JBI critical appraisal checklist | 7/9 (78%) | The study examines a cutaneous anthrax outbreak in West Bengal, tracing transmission to contact with a dead bullock, predominantly affecting males with a significant case fatality rate. Biases include a narrow geographic focus and potential underreporting, limiting broader epidemiological insights |
| Reddy, R et al. 2012^[30]^ | Cohort study | JBI critical appraisal checklist | 7/11 (64%) | The study details a cutaneous anthrax outbreak in Chittoor, India, associated with handling dead livestock. Potential biases include reliance on self-reported activities for identifying risk factors and possible under-reporting due to limited clinical and laboratory confirmation. |
| Bhattacharya et al. 2013^[31]^ | Case report | JBI critical appraisal checklist |  | The study describes a case of cutaneous anthrax in West Bengal, diagnosed through laboratory confirmation of Bacillus anthracis after contact with a sick cow. The reliance on a single case may limit the study's broader applicability. |
| Iqbal N et al. 2015^[33]^ | Case report | JBI critical appraisal checklist | 7/8 (88%) | The study details a fatal case of gastrointestinal anthrax in South India, marked by sepsis and complex symptoms that masked the underlying condition. The reliance on a single, late-stage case may limit the generalizability of the findings. |
| Mondal , TK et al. 2015^[34]^ | Cross sectional study | JBI critical appraisal checklist | 6/9 (68%) | The study investigates a suspected anthrax outbreak in West Bengal, noting high risk among those who handled and consumed meat from a slaughtered animal. The outbreak's characterization relies heavily on clinical symptoms without definitive laboratory confirmation, presenting a significant bias in confirming anthrax as the cause. |
| Deb, S et al., 2015^[35]^ | Case report | JBI critical appraisal checklist | 5/8 (62%) | The study describes a case of cutaneous anthrax relies heavily on clinical symptoms without definitive laboratory confirmation. The findings may have limited generalizability due to reliance on a single case. |
| Achar, A et al. 2018^[36]^ | Cross sectional study | JBI critical appraisal checklist | 8/9 (89%) | The study describes a cutaneous anthrax outbreak in India, highlighting underreporting in rural regions. Potential biases arise from its limited regional focus and primarily microbiological confirmation and lacks advanced diagnostic confirmation |
| Balachandrudu B et al. 2018^[37]^ | Case report | JBI critical appraisal checklist |  | The study reports a cutaneous anthrax outbreak in Visakhapatnam's tribal area, confirmed by culture and PCR. Potential biases stem from its focus on a small, specific group, limiting broader applicability |
| Garg N et al 2018^[38]^ | Case report | JBI critical appraisal checklist | 7/8 (88%) | The study describes a fatal anthrax case in Northeast India, highlighting severe neurological symptoms and the need for prompt treatment. It is limited by its reliance on a single case, which may not capture the full spectrum of the disease's manifestations. |
| Kumar, M et al. 2019^[39]^ | Case report | JBI critical appraisal checklist | 3/8 (38%) | The study on an anthrax outbreak in Jharkhand, India, confirmed Bacillus anthracis using PCR. However, the study may be biased as it only included cases with available clinical specimens for PCR and lacks detailed epidemiological data |
| Nayak, P et al. 2019^[40]^ | Case control study | JBI critical appraisal checklist | 7/10 (70%) | The study on a cutaneous anthrax outbreak in Odisha identified risk factors like consuming or handling meat from ill animals. It suggests biases may arise from recall errors in case-control studies and limited laboratory capabilities for anthrax detection. |
