# Supplementary material for: Epidemiology of Human and Animal Anthrax in India, 1990–2022: A Comprehensive Analysis of Literature and National Surveillance Data
Source: Biomed Res Int. 2025 Dec 21;2025:5633425. doi: 10.1155/bmri/5633425 (PMC12719797; doi:10.1155/bmri/5633425)
Supplement: Supplementary file 2 — Supporting Information 2 Figure S1: Overall district‐wise distribution of animal and human anthrax outbreaks in India, 1990–2022. Figure S2: Death rates due to anthrax in small and large ruminants per 100,000, 2003–2022. (a) Animal anthrax death rates in east. (b) Animal anthrax death rates in south. Death rates for cattle (blue), sheep (red), and goat (black), and the 95% confidence intervals are represented. Figure S3: Strategic approach to establish capability and improve anthrax prevention and control in endemic regions in India. [file BMRI-2025-5633425-s004.docx]

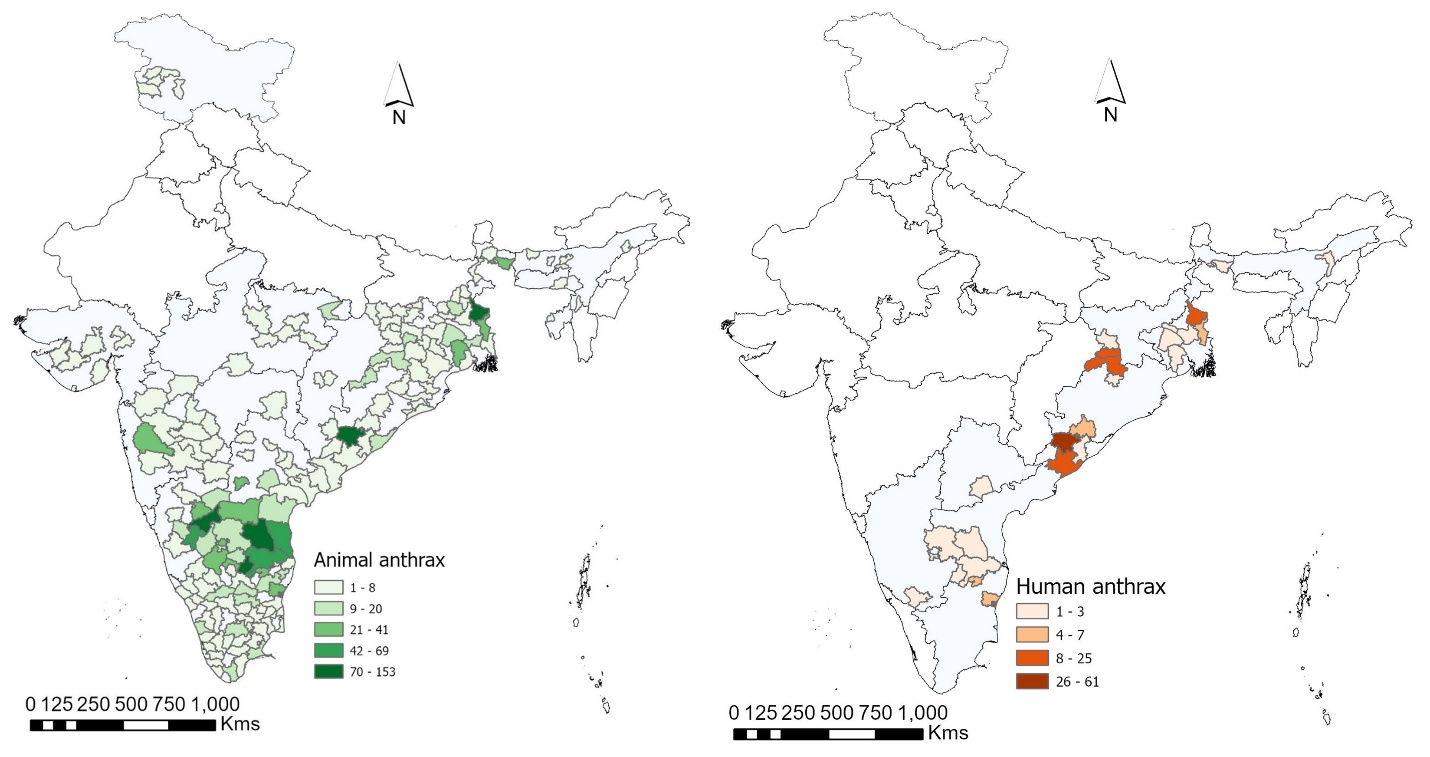


**Supplemental Fig 1. Overall district-wise distribution of animal and human anthrax outbreaks in India, 1990-2022. (A)** Animal anthrax reporting districts. **(B)** Human anthrax reporting districts. Animal outbreaks are represented in shades of green, and human outbreaks are represented in shades of orange.

**
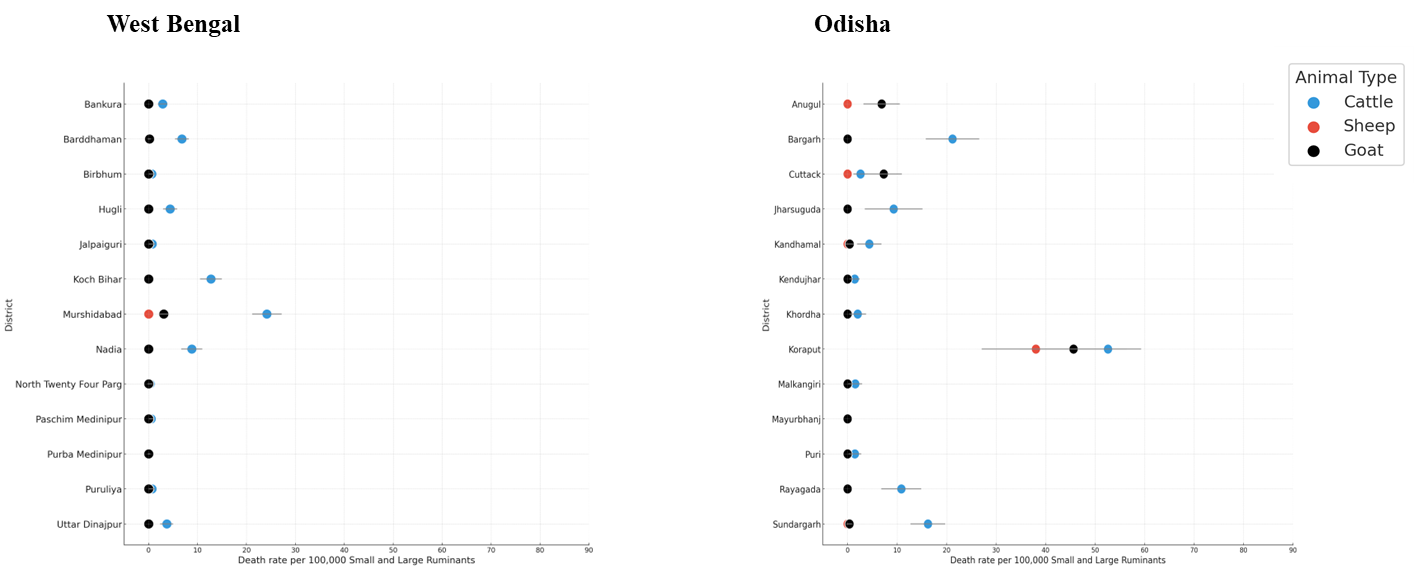
**

A

**
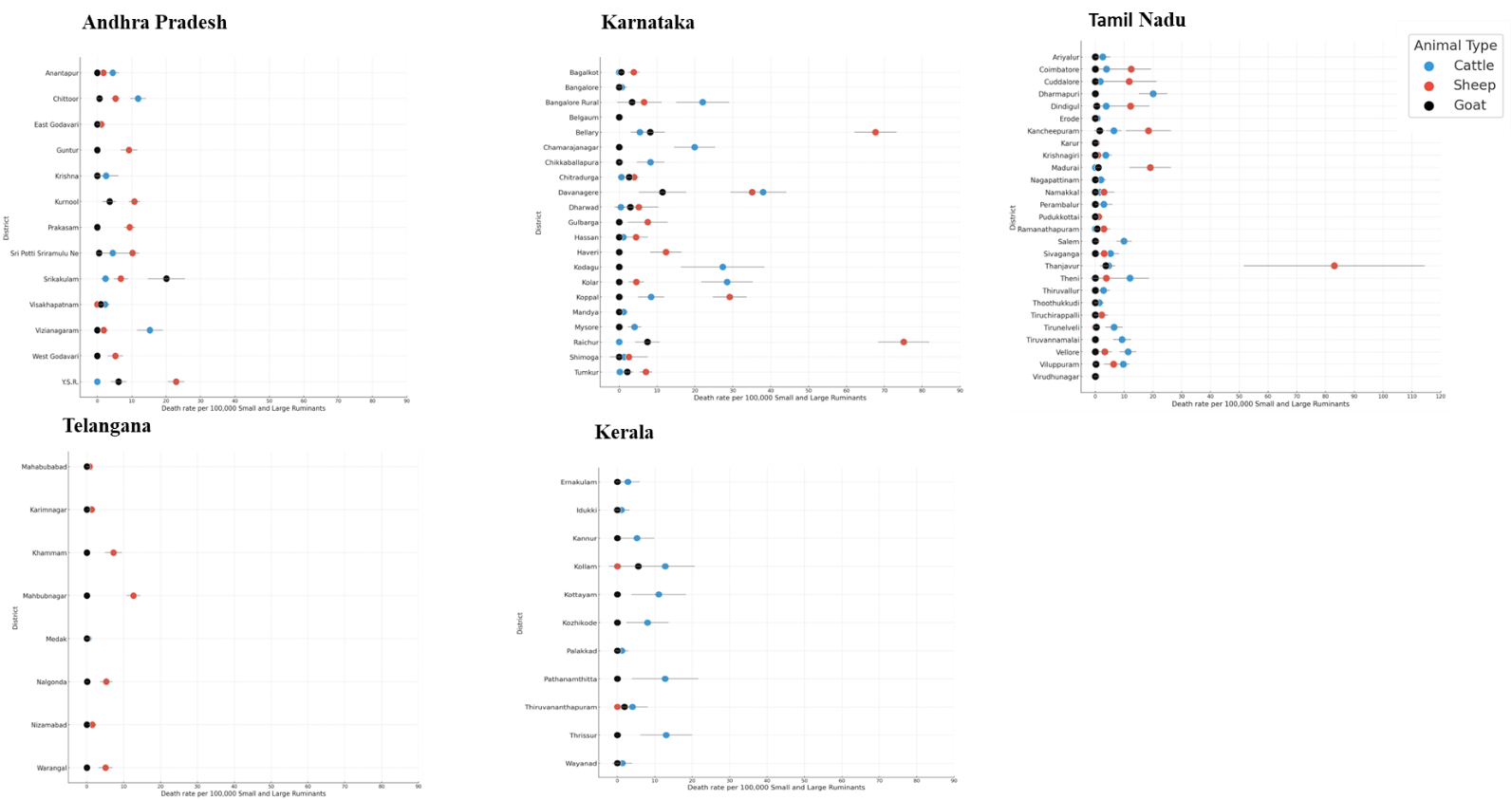
**

B

**Supplemental Fig 2. Death rates due to anthrax in small and large ruminants per 100,000, 2003-2022. (A) Animal anthrax death rates in East. (B) Animal anthrax death rates in South.** Death rates for Cattle (blue), Sheep (red), and Goat (black), and the 95% Confidence Intervals are represented


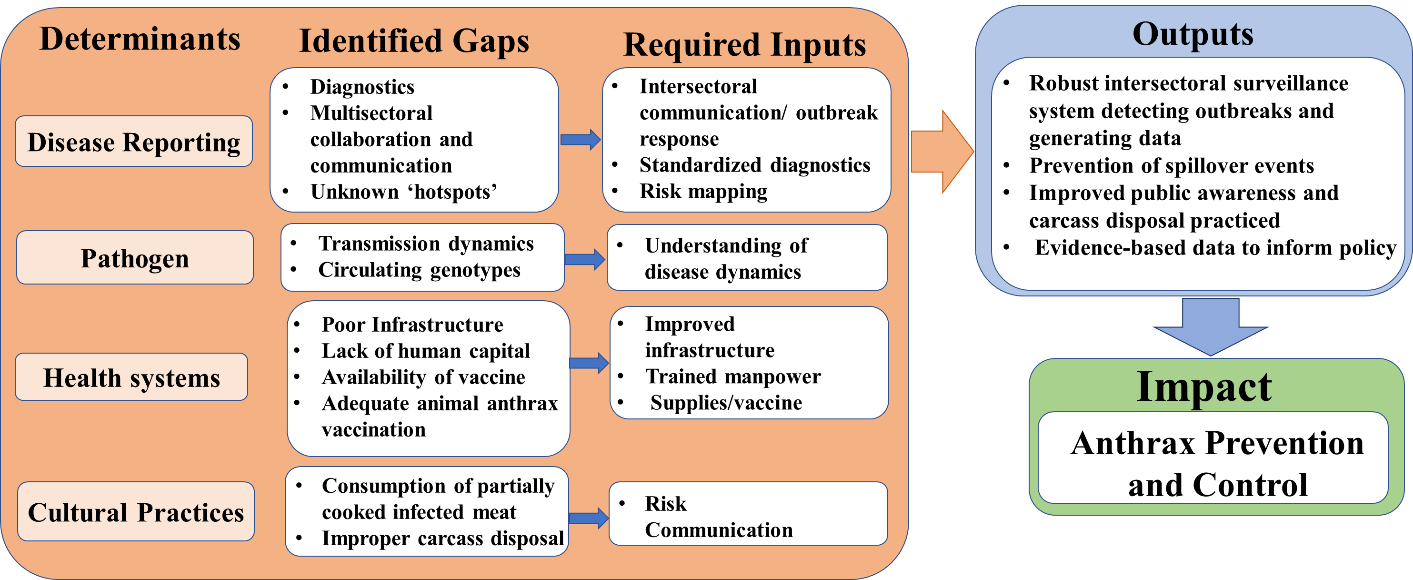


**Supplemental Figure 3: Strategic approach to establish capability and improved anthrax prevention and control in endemic regions in India.**
